# Raman Gas Analysis with External Power Build-Up Cavity of Line-Narrowed 407-nm Laser Diode

**DOI:** 10.3390/s25154600

**Published:** 2025-07-25

**Authors:** Zhongyi Yao, Xinbing Wang, Duluo Zuo

**Affiliations:** Wuhan National Laboratory for Optoelectronics, Huazhong University of Science and Technology, Wuhan 430074, China; d202080953@hust.edu.cn (Z.Y.); xbwang@hust.edu.cn (X.W.)

**Keywords:** Raman gas analysis, power build-up cavity, laser diode, optical feedback, laser linewidth narrowing

## Abstract

An external power build-up cavity of a line-narrowed 407-nm laser diode for Raman gas analysis was demonstrated to possess good gas detection capabilities. By employing an ordinary laser diode without anti-reflection coating or and a bandpass interference filter in an external cavity resonance, the laser linewidth was narrowed by resonant optical feedback, and tens of watts of external cavity power were built up. The coupling mechanism between the semiconductor laser and the external cavity are discussed, as well as the noise background in the experimental results. The Raman spectrum of ambient air was analyzed, achieving a methane detection limit of 1 ppm.

## 1. Introduction

Gas analysis is applied in various fields, such as natural gas extraction [1], industrial process control [2], and environmental protection [3]. Common methods for gas analysis include gas chromatography [4] and infrared absorption spectroscopy [5,6]. The application of gas chromatography is limited by its large size and complex processes. Infrared absorption spectroscopy, which is based on vibrational level transitions of molecules, has the advantage of high sensitivity. However, it requires expensive optical equipment and multiple tunable lasers to detect multiple absorption peaks [7]. Moreover, homonuclear diatomic molecules like O_2_ and N_2_ cannot be detected by infrared absorption spectroscopy [8].

Spontaneous Raman spectroscopy offers advantages such as a convenient process, non-destructive detection, and the simultaneous detection of multiple gas components. Due to the small Raman cross-section of gases, methods for enhancing the Raman scattering are widely adopted. Anti-resonant hollow-core fibers (AR-HCF) have shown good enhancement effects due to their large core area, high transmission efficiency, and low fluorescence background. Bai et al. [9], Knebl et al. [10], Yang et al. [11,12], and Kelly et al. [13] achieved promising results in this regard. In the field of cavity-enhanced techniques, King et al. [14] used an anti-reflectively coated laser and a high-finesse external cavity to achieve an intracavity power of 100 W. Keiner et al. used a similar experimental setup to study the concentration of greenhouse gases in marshlands [15,16] and the gases produced by microbial activity [17]. Ohara et al. [18,19] conducted cavity-enhanced studies and achieved a power build-up of 80 W. Hippler et al. [20] utilized a laser diode in resonance with an external cavity, leveraging the weak feedback principle of the semiconductor laser, which resulted in an enhancement of the light intensity by a factor of 1000. Wang et al. [21] employed a 650-nm single-longitudinal-mode semiconductor laser with a high-finesse external cavity, achieving an external cavity power of 100 W and a limit of detection (LOD) of 17.4 ppm for CO_2_. Yang et al. [22] applied the Pound–Drever–Hall (PDH) technique for cavity enhancement, achieving sub-ppm LODs for various molecules, though this method is costly. In terms of multi-pass cavity enhancement, Petrov et al. [23,24] used a near-concentric cavity with a 5 W laser to achieve an LOD of 0.06 ppm for methane at 20 atmospheres. Wen et al. [25,26] used a multi-pass cavity and collected Raman signals at 90 degrees. They detected the main components of the gases. Velez [27] and Muller et al. [28,29], using a near-concentric cavity and collinear collection methods, achieved LODs of 0.5 ppm and 0.1 ppm for methane, respectively. Wang et al. [30] used a multi-pass cavity and novel polarization filter to achieve an LOD of 6 ppm for CO_2_. Wang et al. [31] achieved an LOD at the sub-ppm level using a Z-shaped multi-pass cavity.

In this article, we use a laser diode (LD) without anti-reflection (AR) coating or an external cavity for resonance enhancement, which are easily obtainable. We explored mode matching between the laser and the external cavity and compressed the laser linewidth to improve spectral resolution. By these means, we achieved an external cavity build-up power of 14 watts and detected the methane in the air (2 ppm), reaching an LOD in the ppm range.

## 2. Experimental Setup

**Figure 1:**
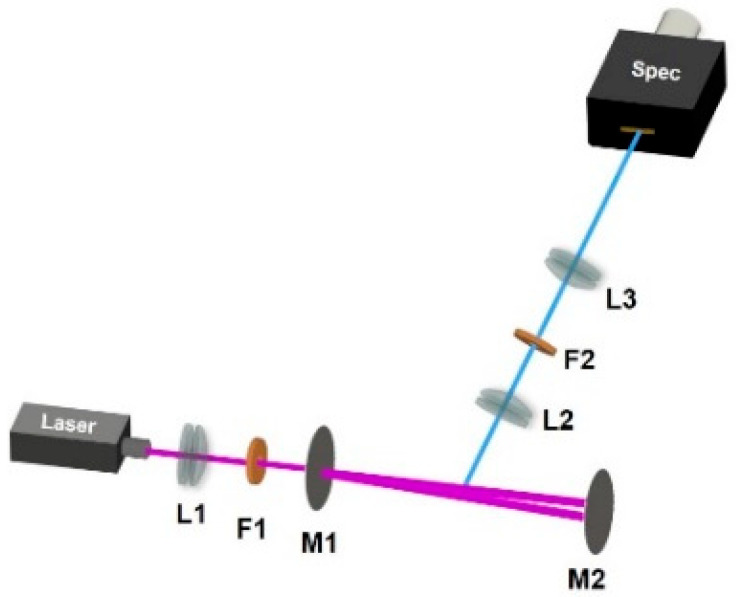
Experimental setup for Raman measurements. L1 = aspheric lens; L2 & L3 = achromatic lens; F1 = bandpass filter; F2 = long-pass filter; M1 & M2 = concave mirror.

The cavity-enhanced experimental setup is illustrated in Figure 1. A 407-nm multimode LD (700 mW) serves as the excitation source, which is a standard one without AR coating. L1 (C775TMD-405, f = 4.0 mm, Thorlabs, Newton, NJ, USA) is an aspheric lens, which is used to achieve mode matching between the diode laser and the confocal cavity. The external resonant cavity is a symmetric confocal resonator consisting of two mirrors with a curvature radius of 50 mm and a separation distance of 50 mm. The reflectivity of M1 at 407 nm is 96.5%, while M2 has a reflectivity of 99.5%. From the reflectivity and the length of the cavity, the linewidth of the cavity is calculated as 43.8 MHz. The cavity axis (line connecting the two centers of the spherical mirrors) is slightly angled relative to the incident laser beam, creating a V-shaped beam path inside the cavity. The beam within the cavity reflects to the LD and forms optical feedback. When the laser is controlled by the optical feedback and oscillates at the resonant frequencies of the cavity, resonance occurs, enhancing the laser power within the cavity. The direct reflection of the laser incident on M1 is reflected away and does not interfere with the optical feedback. A bandpass filter F1 (FF01-405/10-25, or LL01-407-12.5, Semrock, Rochester, NY, USA) is placed behind the coupling lens L1 with two main functions: firstly, to eliminate spontaneous emission from the laser and other interfering light, and secondly, to narrow the linewidth of the resonant cavity system.

Raman radiation is collected at a 90° angle to the laser beam. To increase the signal level, the slit of the imaging spectrometer is set parallel to the laser beam inside the cavity, increasing the collection volume of the Raman radiation. The Raman radiation is collected by an achromatic lens L2 (f = 40 mm) and passes through a long-pass filter F2 (BLP01-405R-25, Semrock). The Raman radiation then enters the spectrometer through a lens L3. The spectrograph is a custom-built transmission-type volume-phase grating spectrograph with a slit width of 10 µm and a resolution of 5 cm^−1^. The F# of the spectrometer is 1.8, and the attached CCD detector to the spectrograph is PIXIS-400B from Princeton Instruments (Trenton, NJ, USA), in which the height of the active area is 8 mm. Using these parameters, the collection area of the Raman analysis system can be estimated as 8 μm × 4 mm. Mechanical structures were used to block stray light, and the experiments were conducted in a dark environment.

## 3. Results and Discussion

### 3.1. Mode-Matching of the Laser Beam to the External Cavity

Optical feedback external cavities have been extensively applied to research. There are mainly two types of cavity geometries: co-axial coupling geometry [14,32,33,34,35] and off-axial (V-shaped) coupling geometry [36,37,38,39]. When utilizing an AR-coated laser diode, precise mode matching between the LD and the external cavity is not essential. In this case, resonance can still be maintained despite variations in the separation between the LD and the external cavity. For example, even when the external cavity is displaced by a small distance from the mode-matching position, stable resonance can still be achieved [14].

V-shaped coupling geometry was utilized in our work as the applied LDs were standard ones with no AR coating. In this way, when the mode is matched, the distance between the external cavity and the laser must remain unchanged. If the external cavity is repositioned, the coupling lens must be adjusted accordingly; otherwise, the resonance will be lost.

The axis of the power buildup cavity (PBC) was only slightly tilted with the incident beam; thus, the Raman scattered radiation from the two branches of the V-shaped beam trajectory was collected. As there was only a small angle between the two branches, the V-shaped coupling to the PBC can be approximated by coaxial coupling to make the discussion easier. In the symmetric confocal cavity, the Gaussian beam forms degenerate transverse modes. There are three scenarios in which a Gaussian mode can be self-reproduced after a round trip in the cavity [40]:

1. A symmetrically distributed Gaussian beam, with the beam waist located at the center of the cavity. In this case, the radius of beam waist can be calculated as $w_{0}=\sqrt{L\lambda /2\pi }$, where L is the cavity length and $w_{0}$ is the beam waist. Inside the cavity, the wavefronts of the forward and backward beams are completely overlapped and coincide with the mirror surfaces at the position of the cavity mirrors.

2. The position of the beam waist is deviated from the cavity center and $w_{0}<\sqrt{L\lambda /2\pi }$. Here, the wavefronts of the forward and backward beams still perfectly overlap and coincide with the mirror surfaces.

3. A round-trip self-reproducing Gaussian beam, where the wavefronts of the forward and backward beams do not fully overlap, and the wavefronts at the cavity mirrors do not coincide with the mirror surfaces. However, after a round trip, the beam can be reproduced.

In the first two cases, the backward-propagating beam in the PBC can return to the laser with the same q parameter as the diode laser mode. In the third case, however, the backward beam cannot return to the laser because its spot does not coincide with the forward beam, and the optical feedback cannot be established.

**Figure 2:**
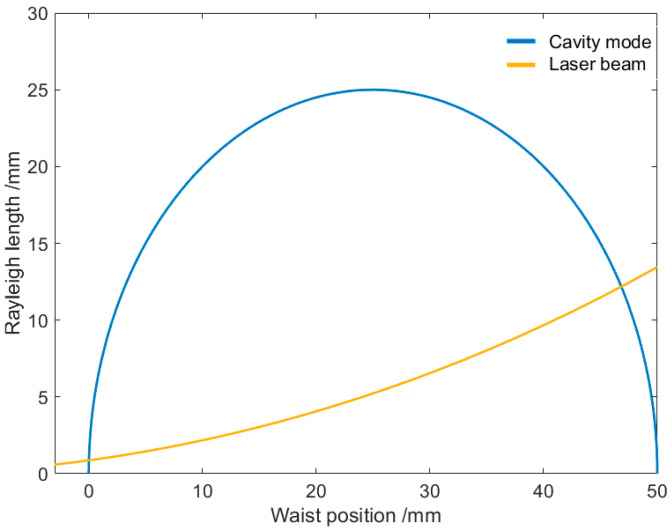
Mode-matching points determined by the intersection of curves of Rayleigh length vs. waist position of PBC Gaussian modes and the incident beam. The curve for the incident beam is a result of aligning the aspheric lens L1 along the beam.

The mode matching in this study was achieved corresponding to the 2nd scenario. In this scenario, as well as the 1st scenario, the relation of Rayleigh length $z_{0}$ of the Gaussian modes inside the PBC with the waist position can be expressed as $z_{0}=\sqrt{x\left(L-x\right)}$ where x is the distance to M1. This relationship is shown in Figure 2 as the blue line taking L=50 mm according to the experimental setup, which can also be shown in another way as $w_{0}$ vs. x, calculating the beam waist $w_{0}$ from the Rayleigh length with the equation $z_{0}=\pi w_{0}^{2}/\lambda$. During the experiment, the positions of the laser diode and the PBC are fixed, and the mode-coupling is achieved by aligning the position of aspherical lens L1. When the position of L1 is changed, the position of the beam waist of the output beam (the incident beam to the PBC) is also changed. The relation of the Rayleigh length of the incident beam with the waist position is also shown in Figure 2 as the yellow line. The intersection points of these two curves are the positions for mode matching. The second one corresponds to the experimental observations, where the beam waist is located on the right side of the external cavity.

**Figure 3:**
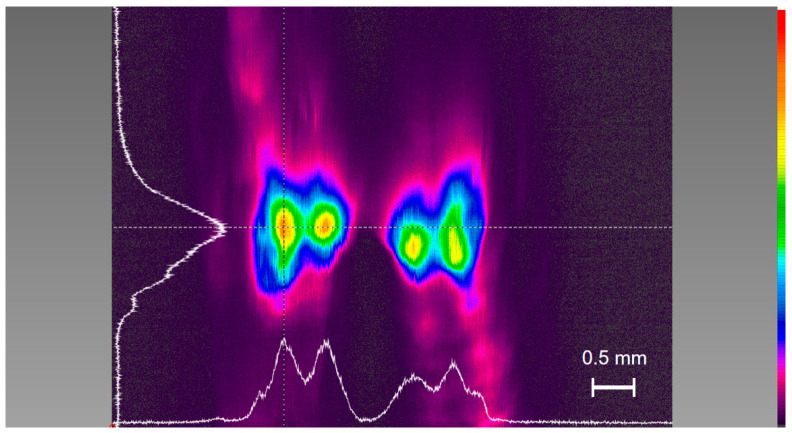
Beam pattern measured after mirror M2. Two output beams can be observed after mirror M2, each containing two intensity peaks.

When a beam profile (SP620U, Newport) was set at a distance of about 50 mm from mirror M2, a beam pattern as shown in Figure 3 was measured, which shows two output beams, each with two peaks.

In the experiment, the intracavity build-up power was estimated to be 20.8 W (the power of a single beam is 10.4 W, and the total power is 20.8 W) based on a reflectivity of 99.5% for M2 and the measured transmitted power 52 mW after M2, in which the power of all forward beams can be calculated as $52/\left(1-99.5\%\right)\approx 10.4$ W. When the LD was coupled to a cavity with two mirrors of reflectivity 96.5%, the transmitted power after M2 was 182 mW, since laser radiation is emitted from both the left and right sides of the PBC simultaneously, from which the output of the external cavity can be estimated as 364 mW and the coupling efficiency can be calculated as 52%. When a single-transverse-mode LD of 150 mW (another laser) was applied, the coupling efficiency was 90%. The coupling efficiency is defined as the ratio of the optical power coupled into the external cavity to the laser power.

### 3.2. Narrowing of the Laser Linewidth

**Figure 4:**
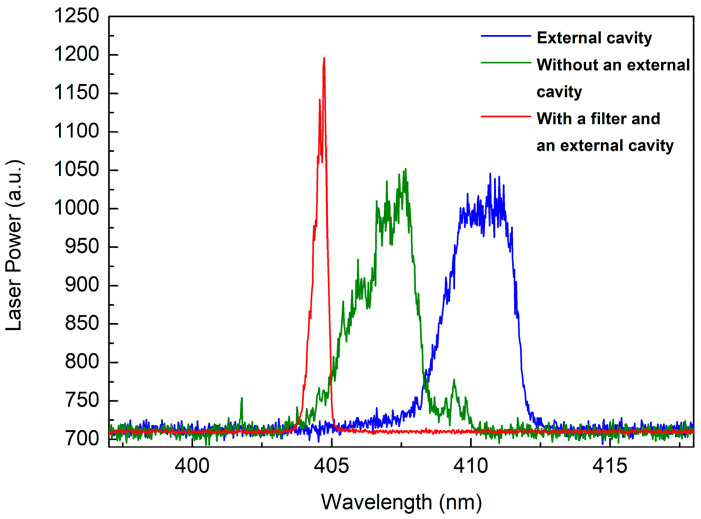
Comparison of the emission spectrum of an LD: (green) free-running LD, (blue) diode with an external cavity, (red) diode with a filter and an external cavity.

Laser diodes have a relatively broad gain spectrum, while the transmission curve of interference filters typically features steep rising and falling edges. When the passband curve of the dielectric film filter intersects with either the rising or falling edge of the LD’s gain profile, the laser will emit at the peak of the filtered gain, and the linewidth narrows [41]. In this study, we conducted two tests using two types of interference filters (FF01-405/10-25 and LL01-407-12.5, Semrock) with bandwidths of 14 nm and 1.5 nm, respectively, both of which were effective. When the laser, interference filter, and external cavity were combined, the bandwidth significantly decreased. Simultaneously, the laser wavelength also shifted. The free-running laser linewidth of the 700 mW LD was 2.7 nm, which remained at 2.7 nm in resonance with the external cavity, but reduced to 0.42 nm, as shown in Figure 4, when a filter was applied. When using a laser with a maximum power of 500 mW (another laser), the free-running laser linewidth was 1.2 nm, which narrowed to 0.9 nm in resonance with the external cavity and further reduced to 0.3 nm with a filter. The spectral shape was random during free-running operation, but it became symmetrical during resonance with the external cavity, possibly due to the increase in cavity length and photon lifetime.

On the other hand, the external cavity power changed after the insertion of the optical filter. Without the filter, the intracavity power was 20.8 W; with the filter inserted, it decreased to 14.1 W, approximately two-thirds of the original value. The filter’s peak transmission is 96%. This power reduction may be due to the lasing wavelength not coinciding with the filter’s peak transmission, instead lying near the edge of its transmission curve. Despite the decrease in power, subsequent Raman spectral measurements showed that the signal intensity with the filter was five times higher than without it. Furthermore, the spectral resolution was significantly improved. These results indicate that the use of a bandpass filter is advantageous.

For gas analysis, the resolution of Raman spectroscopy is determined by both the spectrometer and the laser light source, and the resolution is 20 cm^−1^. Our spectrometer features a narrow slit width, enabling high resolution. When applied in this experiment, the height of the slit is also taken into consideration. Due to the spectrometer’s smile effect, the slit imaging on the CCD forms a curved image, which adversely affects the resolution. In this experiment, the resolution of Raman spectrum exceeds that of the spectrometer itself, primarily due to the laser linewidth.

### 3.3. Raman Spectroscopy of Ambient Air

**Figure 5:**
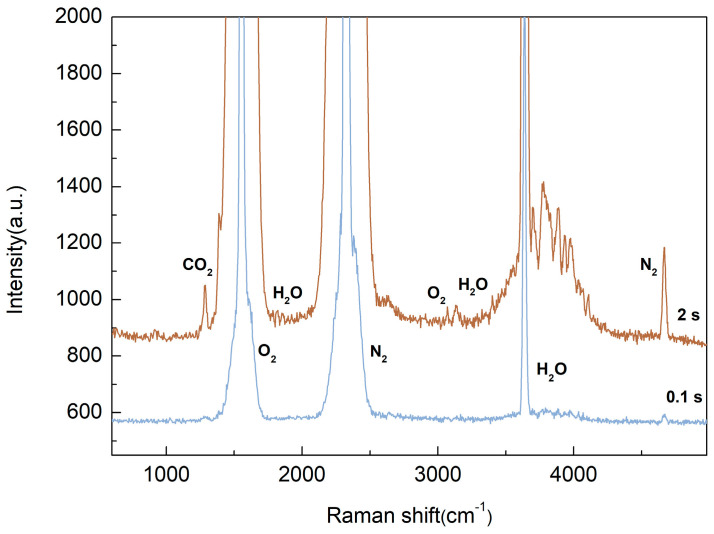
Simultaneous detection of five chemical species found in ambient laboratory air at varying concentrations with exposure times of 0.1 s (blue) and 2 s (red).

Ambient air was used as the sample to demonstrate the capability of our experimental setup. The Raman spectra of ambient air with integration times of 0.1 s and 2 s are illustrated in Figure 5. At an integration time of 0.1 s, CO_2_ (1285 cm^−1^ and 1388 cm^−1^) [42], O_2_ (1556 cm^−1^) [42], N_2_ (2331 cm^−1^) [42], H_2_O (3657 cm^−1^), and the overtone of N_2_ (4656 cm^−1^) [8,25,26] can be detected. At an integration time of 2 s, H_2_O (1768 cm^−1^ to 1880 cm^−1^ and 3130 cm^−1^) [23] and the overtone of O_2_ (3087 cm^−1^) [25] can be observed. These results demonstrate the detection capability of the device. At the Q-branches of nitrogen and oxygen, the CCD is saturated. In the 2-s test, the signal intensities of O_2_ overtone (3087 cm^−1^) and H_2_O (3130 cm^−1^) were comparable, which is related to the ambient humidity on the day of the experiment. When detecting ambient air, the concentrations of CO_2_ and H_2_O are not constant.

**Figure 6:**
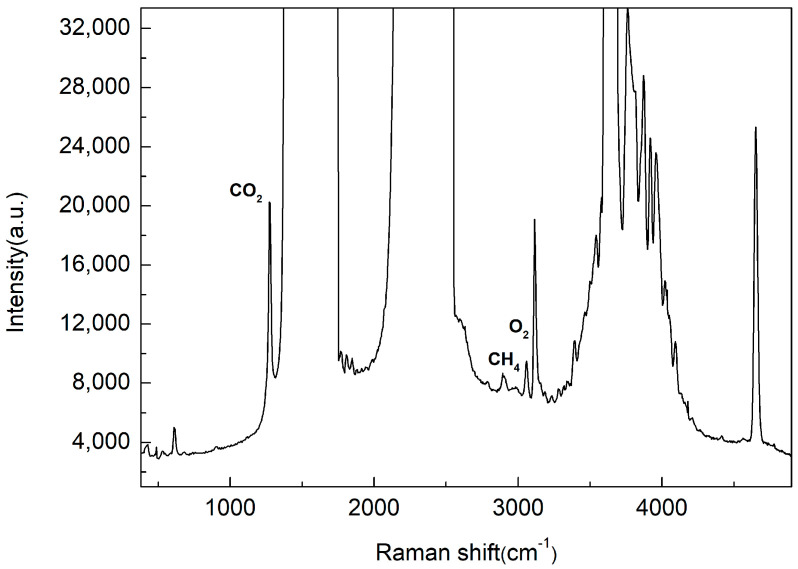
Raman spectrum of atmospheric air. Exposure time is 300 s.

For spontaneous Raman detection, reducing the signal background is crucial. The background consists of detector dark current, stray light from the light source, fluorescence from lenses and mirrors, and ambient light, as well as Raman and fluorescence radiation from the sample under detection. Another Raman spectrum of ambient air is shown in Figure 6, of which the resolution is 20 cm^−1^, the integration time is 300 s, and the spectral range is from 390 cm^−1^ to 4900 cm^−1^. Figure 6 presents the unprocessed spectrum, showing a relatively low background with a maximum of about 7000 counts for such a long integration time, which is advantageous for detecting trace gas components. As shown in Figure 6, CH_4_ (2917 cm^−1^) [42] in ambient air can be detected, with a concentration of about 2 ppm. Methane can also be detected with a shorter integration time of 100 s. The CH_4_ peak (2917 cm^−1^) is located on the wing of the S-branch of the N_2_ band.

The LOD is defined as the partial pressure (or concentration) of a constituent when the signal is three times the noise level (standard deviation of the background). The LOD for CO_2_ was calculated as follows: a blank spectrum near the CO_2_ signal was selected as the baseline, by which the standard deviation was calculated. The calculated LOD for CO_2_ was estimated to be 6.5 ppm with the concentration of ambient CO_2_ set at 700 ppm, a calculated value derived from the literature [23,27]. The ratio of the Raman scattering cross-section of carbon dioxide to methane is 1.1:8.6; the LOD for methane was estimated as 0.9 ppm.

In the study of Keiner et al. [17], the laser linewidth was not narrowed. Our experiment enhanced both resolution and detection capability. In the research conducted by Petrov et al. [23], a 5-W laser and a multi-pass enhancement cavity were utilized. By collecting Raman signals at a 90° angle, they achieved an LOD of 1.2 ppm for methane. In our study, we employed a 700-mW laser and obtained an LOD comparable to theirs. In studies of resonant cavity Raman spectroscopy, research groups [21] generally prefer to collect signals at a 0°, which usually results in good detection sensitivity. However, our study shows that collecting at a 90° angle is also a viable option, capable of attaining a relatively low LOD.

**Figure 7:**
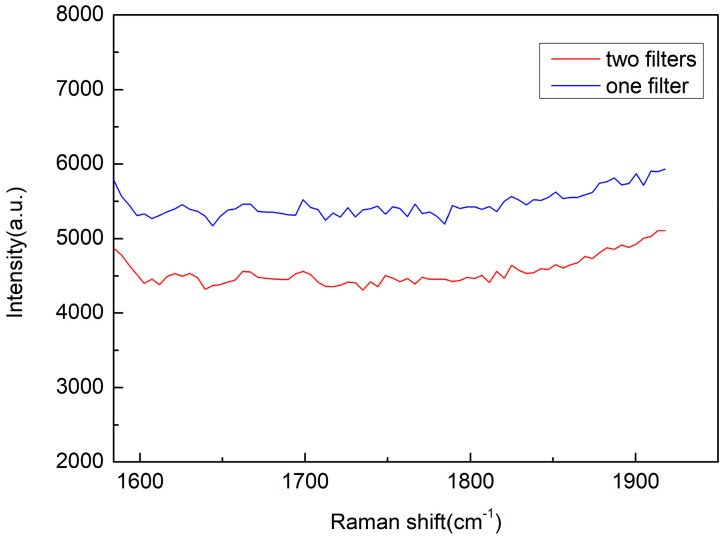
Blue line represents background signal using one filter, while red line indicates results with two filters.

To further reduce the background, we investigated its sources. Diode lasers exhibit strong spontaneous emission, which has a broad spectral range and aligns with the laser’s direction. Two experiments were conducted. In the first experiment, only one band filter at the laser wavelength was used to remove stray light. In the second experiment, two filters (FF01-405/10-25, and LL01-407-12.5, Semrock) were applied simultaneously. The results are shown in Figure 7, in which the blue line represents the noise background using one filter, while the red line indicates the results with two filters. The laser powers set in these experiments were similar. The overtone peak intensity of nitrogen (4656 cm^−1^) before and after adding another optical filter was 5342 and 5844 (relative altitude of the signal), respectively, and the background intensity was 5299 and 4455. Normalizing the background to the N_2_ overtone signal peak, the background intensity becomes 5299 and 4072. This indicates that the background has decreased by 1227 (23%) in counts. It can be observed that after adding the filter, the background noise decreased. However, the decrease in background signal is not significant, suggesting there are other sources of noise.

## 4. Conclusions

Improving the LOD is essential for the analysis of trace gaseous constituents. To this end, a cavity-resonance enhancement of Raman scattering was investigated using an external cavity and a 407-nm blue–violet LD without AR coating—both readily accessible experimental components. A bandpass filter was inserted between the coupling lens and the PBC, which narrowed the laser linewidth to 0.3 nm, contributing to improved spectral resolution. Using this configuration, an external cavity build-up power of 14 W and an LOD of 1 ppm for CH_4_ were achieved, demonstrating the effectiveness of cavity-enhanced Raman spectroscopy with a blue–violet LD. By applying two bandpass filters simultaneously, the background was reduced by 23%, indicating that spontaneous emission is a significant contributor to the background signal.

## Data Availability

The data supporting the findings of this study are available from the corresponding author upon reasonable request.
